# Neural mechanisms of symptom improvements in generalized anxiety disorder following mindfulness training^☆^^☆☆^

**DOI:** 10.1016/j.nicl.2013.03.011

**Published:** 2013-03-25

**Authors:** Britta K. Hölzel, Elizabeth A. Hoge, Douglas N. Greve, Tim Gard, J. David Creswell, Kirk Warren Brown, Lisa Feldman Barrett, Carl Schwartz, Dieter Vaitl, Sara W. Lazar

**Affiliations:** a Massachusetts General Hospital, 120 2nd Ave., Charlestown, MA, 02129, USA; b Bender Institute of Neuroimaging, Justus-Liebig University, Otto-Behaghel-Str. 10H, 35394 Giessen, Germany; c Department of Psychology, Carnegie Melon University, 5000 Forbes Ave., Pittsburgh, PA 15213, USA; d Department of Psychology, Virginia Commonwealth University, 806 West Franklin Street, Richmond, VA, 23284, USA; e Department of Psychology, Northeastern University, Boston, MA, 02115, USA

**Keywords:** Generalized anxiety disorder, Emotion regulation, Mindfulness, Intervention, Longitudinal, Amygdala, Prefrontal cortex, Connectivity, Ventrolateral prefrontal cortex, Beck Anxiety Inventory, Stress

## Abstract

Mindfulness training aims to impact emotion regulation. Generalized anxiety disorder (GAD) symptoms can be successfully addressed through mindfulness-based interventions. This preliminary study is the first to investigate neural mechanisms of symptom improvements in GAD following mindfulness training. Furthermore, we compared brain activation between GAD patients and healthy participants at baseline. 26 patients with a current DSM-IV GAD diagnosis were randomized to an 8-week Mindfulness Based Stress Reduction (MBSR, N = 15) or a stress management education (SME, N = 11) active control program. 26 healthy participants were included for baseline comparisons. BOLD response was assessed with fMRI during affect labeling of angry and neutral facial expressions. At baseline, GAD patients showed higher amygdala activation than healthy participants in response to neutral, but not angry faces, suggesting that ambiguous stimuli reveal stronger reactivity in GAD patients. In patients, amygdala activation in response to neutral faces decreased following both interventions. BOLD response in ventrolateral prefrontal regions (VLPFC) showed greater increase in MBSR than SME participants. Functional connectivity between amygdala and PFC regions increased significantly pre- to post-intervention within the MBSR, but not SME group. Both, change in VLPFC activation and amygdala–prefrontal connectivity were correlated with change in Beck Anxiety Inventory (BAI) scores, suggesting clinical relevance of these changes. Amygdala–prefrontal connectivity turned from negative coupling (typically seen in down-regulation of emotions), to positive coupling; potentially suggesting a unique mechanism of mindfulness. Findings suggest that in GAD, mindfulness training leads to changes in fronto-limbic areas crucial for the regulation of emotion; these changes correspond with reported symptom improvements.

## Highlights

• GAD patients show higher amygdala activation to neutral faces than healthy subjects.
• In GAD patients, amygdala activation decreased after stress reduction interventions.
• Ventrolateral PFC activation during affect labeling increases following mindfulness.
• Amygdala–PFC functional connectivity increases following mindfulness.
• These changes following mindfulness correlate with improvements in anxiety symptoms.

## 1 Introduction

Generalized anxiety disorder (GAD) is characterized by pervasive and intrusive worry (American Psychiatric Association, 2000), and is associated with impairment in daily functioning. Individuals with GAD show deficits in emotion regulation (Tull et al., 2009), such as a greater negative reactivity to, and poorer understanding of emotions (Mennin et al., 2005). Psychological treatments therefore aim to help clients to become more comfortable with arousing emotional experiences and foster better emotion regulation (Mennin et al., 2002). Mindfulness-based interventions, which focus on the cultivation of attention to present moment experiences with an attitude of openness and non-judgmental (Bishop et al., 2004; Kabat-Zinn, 1990), directly address such deficits. They have been shown to effectively ameliorate anxiety symptoms (Hofmann et al., 2010), and have been successfully applied in the treatment of GAD (Hoge et al., in press; Roemer et al., 2008). While mindfulness-based interventions are increasingly applied in the therapeutic context (Baer, 2003; Grossman et al., 2004), the investigation of the neurobiology underlying the beneficial effects is still in its infancy (Davidson et al., 2003; Farb et al., 2010; Gard et al., 2012; Goldin and Gross, 2010; Goldin et al., 2012; Hölzel et al., 2010). To date, the neural mechanisms underlying the effects of mindfulness-based interventions on GAD have not been studied.

Models of various anxiety disorders hypothesize amygdala hyperresponsivity to threat-related stimuli (Etkin and Wager, 2007; Rauch et al., 2003). However, it has not been unambiguously established how brain activation in response to evocative stimuli differentiates GAD patients from healthy participants (Etkin, 2011). A few GAD studies have found that consciously presented threatening stimuli (posed facial expressions) do not evoke amygdala hyperactivation (Blair et al., 2008; Monk et al., 2006; Palm et al., 2011; Whalen et al., 2008). In one study, viewing posed angry faces was even associated with amygdala hypoactivation in these patients (Blair et al., 2008). However, adolescents with GAD showed exaggerated amygdala activation in response to nonconsciously presented angry faces (Monk et al., 2008), and adult GAD patients show greater amygdala activation during anticipation of seeing aversive or neutral pictures (Nitschke et al., 2009) suggesting that GAD patients may be more sensitive to ambiguous stimuli than to overtly threatening stimuli.^2^

Anxiety symptoms have also been associated with abnormalities in prefrontal activation and altered relationships between activity of prefrontal regions and amygdala (Kim et al., 2011a, 2011b). For example, stronger activation of the ventrolateral prefrontal cortex (VLPFC) in response to angry faces has been reported in GAD patients as compared to healthy controls, and greater VLPFC activation has been associated with less severe anxiety in these patients (Monk et al., 2006). There is speculation that enhanced VLPFC activation in GAD patients serves a compensatory response designed to regulate abnormal function (Monk et al., 2006). Interestingly, treatment of GAD with selective serotonin reuptake inhibitors (SSRIs) or cognitive behavioral therapy (CBT) has been shown to increase VLPFC activation (Maslowsky et al., 2010). These findings suggest that increased VLPFC activation in GAD is part of a compensatory mechanism that can be enhanced by treatment. The VLPFC is involved in inhibitory control (Cohen et al., 2013) and its activation typically increases when healthy subjects voluntarily downregulate unpleasant emotions (Ochsner et al., 2004; Phan et al., 2005; Wager et al., 2008). It modulates amygdala responses during strategic emotion regulation processes (Hariri et al., 2003; Ochsner and Gross, 2005), and it has been speculated that breakdowns in amygdala–VLPFC interactions might influence anxiety (Hariri et al., 2003). GAD patients show weaker coupling between the VLPFC and amygdala than healthy controls (Etkin et al., 2009; Monk et al., 2008), suggesting modified connectivity between these two regions. Other prefrontal regions, too, have shown abnormal – decreased and increased – connectivity with the amygdala in GAD (Etkin et al., 2009) and in heightened state anxiety (Kim et al., 2011b).

Labeling the affect of encountered stimuli, such as facial expressions, has been suggested to be an incidental emotion regulation process that helps attenuate distress (Lieberman et al., 2011) and to diminish anxiety in a clinical context (Kircanski et al., 2012; Tabibnia et al., 2008). Several fMRI studies have reported that explicitly labeling an evocative stimulus can lead to reduced amygdala response (Foland et al., 2008; Hariri et al., 2000; Lieberman et al., 2007) and increase activation in the VLPFC (Lieberman et al., 2007). Interestingly, in a study on the neural correlates of trait mindfulness, Creswell et al. found that higher trait mindfulness was related to greater activation of prefrontal areas, including ventrolateral and medial regions, lower amygdala activation, and greater amygdala–prefrontal connectivity during affect labeling (Creswell et al., 2007), suggesting a potential neural mechanism of mindfulness training. Given the implication of these same neural regions in GAD, we investigated whether prefrontal and amygdala activation and amygdala–prefrontal connectivity would be modified during affect labeling in GAD patients following mindfulness training. Since mindfulness works through enhanced recognition of emotional states, the affect labeling task was chosen in order to expose the beneficial effects of this training.

The present randomized trial is an initial investigation of neural mechanisms underlying GAD symptom improvements following an eight-week mindfulness-based stress reduction (MBSR (Kabat-Zinn, 1990)) program relative to a structurally equivalent, active control intervention. We measured brain activity with fMRI during the explicit labeling of posed emotional expressions (neutral and angry) both before and after intervention in both GAD groups and in comparison to a reference group of healthy controls. We explored whether 1) GAD patients would show altered amygdala responses to angry and neutral faces compared with healthy participants, 2) GAD patients receiving the MBSR intervention would show greater attenuation in amygdala response compared to the control intervention, 3) GAD patients receiving MBSR would show a greater increase of prefrontal activation, as well as stronger increases in amygdala–prefrontal functional connectivity, compared to the control intervention, and 4) changes in brain activation and functional connectivity would be related to reduced anxiety symptoms.

## 2 Methods

### 2.1 Participants

29 GAD patients were recruited to participate in the MRI study. Participants were assigned to either the MBSR program or the active control intervention, the stress management education (SME) program, based on the time of enrolment into the study (block randomization); 15 were allocated to the MBSR group, and 14 to the SME group. Three subjects in the SME group dropped out (one moved out of town, one had a panic attack in the scanner, and one did not complete the class). Complete data sets were thus available from 26 GAD patients. Furthermore, 26 healthy demographically matched individuals were included for baseline comparisons.

GAD patients were recruited from among participants of a larger RCT (Hoge et al., in press). Healthy participants were recruited through local newspapers and email lists advertising a stress-reduction intervention. All participants were right-handed, had no significant previous meditation experience (≤ 10 sessions) and complied with scanner safety requirements. The groups did not significantly differ in age, gender, and education level (Table 1). All participants were assessed with the Structured Clinical Instrument for DSM-IV (SCID (First et al., 2002)) by a trained clinician. Healthy participants were included if they did not meet any DSM-IV Axis I disorder, and did not take medications that alter cerebral blood flow or metabolism. Patients were included if they met a current DSM-IV GAD diagnosis. Four participants additionally met a diagnosis for comorbid major depressive disorder (MBSR group: 3), one for panic disorder (in the control group), and six for social anxiety disorder (SAD; MBSR group: 5).^3^ Four patients were medicated (MBSR group: 3), three on daily SSRIs, and one taking trazodone twice a week for insomnia. Medicated subjects were included if they had been on a stable dose for at least two months prior to enrollment and agreed to remain on a stable dose over the participation period. Participants provided written informed consent. The study was approved by the Institutional Review Board of Massachusetts General Hospital.

### 2.2 Interventions

Mindfulness Based Stress Reduction (MBSR) is an eight-week, manualized program that was designed specifically to increase mindfulness (Kabat-Zinn, 1990). The program was developed to inculcate emotion regulatory skills to prevent stress and associated mental health problems. It consists of once-weekly, teacher-led group meetings (with duration of two hours in the current study) plus one “day of mindfulness” in the sixth week of the course. During these group sessions, mindfulness is trained via sitting and walking meditation, yoga exercises, and the “body scan”, in which attention is sequentially directed through the whole body. Participants also receive stress education. In addition to the group sessions, participants are instructed to practice mindfulness exercises at home (with the help of an audio recording). They are taught to practice mindfulness also in their daily activities, such as eating, washing the dishes, taking a shower, etc., as a way to facilitate the transfer of mindfulness into daily life.

The stress management education (SME) program was designed as an active control stress reduction intervention for MBSR to disentangle the effects of the mindfulness practice from other, potentially effective elements of the group program, such as a supportive social environment, instructor attention, participants' expectations, and physical exercise. The course has the same in-class and home exercise time as the MBSR program, including a ‘day of stress reduction’ in the 6th week, and is composed of several elements that match the MBSR components. Gentle physical exercises match the yoga component and nutrition and healthy lifestyle education components match the stress education component of MBSR. The SME program is described in detail elsewhere (Hoge et al., in press).

The MBSR group showed greater average compliance with home practice assignments (average = 1116 min, SD = 499 min) than the SME group (average = 868 min, SD = 413 min), but this difference was not significant (*t*(24) = − 1.34, p = .19). There was also no group difference in the number of classes attended (MBSR average = 7.27, SD = .59; SME average = 7.00, SD = .76; *t*(24) = − 1.00, p = .33). Finally, there was no group difference in the number of participants who missed the retreat day (MBSR: 4 out of 15; SME: 1 out of 11; Fisher's exact test, p = .274).

### 2.3 Experimental paradigm

Healthy and GAD participants underwent MRI scanning at a baseline timepoint (‘pre’ intervention), and GAD patients completed the same MRI procedures following the interventions (‘post’). While undergoing fMRI scanning, participants labeled the affect of photographs of angry, happy, and neutral facial expressions from a standardized set (Tottenham et al., 2009). The BOLD response to viewing angry, neutral, and happy faces was compared to a fixation cross control condition. Within each of five blocks, nine pictures of each emotional category were presented in random order and were displayed for four seconds each with varying inter-stimulus intervals. Each face was displayed only once to avoid repetition and familiarity effects. Additional fixation cross resting blocks of 20 s were included after each block. Participants indicated facial affect (affect labeling) by choosing from a pair of labels (e.g., the words ‘angry’ and ‘neutral’) shown below the target face with a button press response. Of note, participants were not instructed to be mindful during the task. The experiment also included a condition wherein subjects labeled the gender of the face. This condition was used only to assess comparability of our baseline findings of healthy participants (comparison affect vs. gender labeling) with previous findings from the field of affect labeling research. For all other analyses, gender labeling conditions were not included, rather contrasts were calculated with the fixation cross condition. This approach was chosen because a) the longitudinal approach already provides a control contrast, where each individual's post data is compared to their own baseline measure, b) responses to gender labeling might also change with the interventions, thereby introducing unnecessary confounds, and c) some subjects reported mentally noting both gender and affect during the gender labeling condition, potentially introducing bleed-over confounds. The scans took place within three weeks before and after the intervention period. The groups did not significantly differ in amount of time between scanning sessions (MBSR mean = 60.3 days, SD = 4.4 days; SME mean = 63.5 days, SD = 6.8 days; independent samples *t*-test: *t*(24) = 1.46, p = .16).

### 2.4 Self-report data

The Beck Anxiety Inventory (BAI (Beck and Steer, 1993)) is a widely used 21-item multiple choice questionnaire assessing anxiety severity. GAD patients completed the BAI before and after the intervention. Results regarding symptom improvements measured with the BAI have been reported elsewhere (Hoge et al., in press). Here, we report these scores as they relate to the imaging findings.

The 10-item version of the Perceived Stress Scale (PSS) is a validated measure assessing the level of subjective life stresses (Cohen and Williamson, 1988; Cohen et al., 1983). Complete data sets were obtained from 25 healthy and 26 GAD participants. Questionnaire data were analyzed with SPSS (SPSS, 2004).

### 2.5 fMRI data acquisition and analysis

Data were acquired with a Siemens Magnetom Avanto 1.5 Tesla MRI scanner (Siemens Medical Systems, Erlangen, Germany). Structural images of the whole brain were collected using a T1 weighted MPRAGE-sequence, consisting of 128 sagittal slices (1.0 × 1.0 × 1.3 mm, TI = 1000 ms; TE = 3.39 ms; TR = 2730 ms). Functional data were acquired across the whole brain using a T2*-weighted gradient echo planar pulse sequence (25 axial slices, 5 mm thickness with no gap, voxel size: 3.1 × 3.1 × 5 mm, TR = 2000 ms, TE = 40 ms, flip angle = 90°, interleaved).

To enable surface-based analysis of fMRI data, anatomical data were processed using FreeSurfer (https://surfer.nmr.mgh.harvard.edu) to construct models of the cortical surfaces for each subject (Dale et al., 1999; Fischl et al., 1999a). These were then registered to a surface-based group template (Fischl et al., 1999b), and aligned with the MNI305 brain (Collins et al., 1995). fMRI data was analyzed with FS-FAST (https://surfer.nmr.mgh.harvard.edu/fswiki/FsFast). Preprocessing involved motion correction using the AFNI (afni.nimh.nih.gov/afni) 3dvolreg program (Cox and Jesmanowicz, 1999), and slice-timing using the FSL (www.fmrib.ox.ac.uk/fsl) slicetimer program. Non-brain voxels were masked out using the FSL Brain Extraction Tool (Smith, 2002). The middle timepoint was registered to the subject's anatomical image using the FreeSurfer bbregister program (Greve and Fischl, 2009). Using this registration, the raw fMRI time series was mapped to the three group analysis spaces: (1) left and (2) right hemisphere group surface space, and (3) MNI305 space within a subcortical mask of gray matter structures. Data was spatially smoothed on the surface by 5 mm full-width/half-max (FWHM) using an iterative technique (Hagler et al., 2006), and in the volume using a 3D smoothing kernel of 5 mm FWHM.

First-level time series analysis was performed using a General Linear Model (Worsley et al., 2002) as implemented in FS-FAST. The hemodynamic response to each experimental condition (neutral, angry, and happy faces, instruction) was modeled using a difference of gamma functions (Glover, 1999). Low frequency drift was accounted for using a 2nd order polynomial and temporal whitening (Burock and Dale, 2000). First-level contrasts were then carried up to the group level random effect analysis. Two masks were created to restrict multiple comparison corrections to: (1) bilateral amygdalae in volume space (Fischl et al., 2002), and (2) frontal cortex in the surface space (excluding the precentral gyrus and paracentral lobule) plus insula, based on the regions defined by Desikan et al. (2006). Exploratory analyses across the whole brain were also performed. For each analysis space (i.e., left and right hemispheres and subcortical areas), we applied a cluster-based correction for multiple comparisons using a simulation-based technique (Hagler et al., 2006; Hayasaka and Nichols, 2003), using a cluster-forming threshold of p < 0.05.

Furthermore, a standard connectivity analysis examined interactions between the chosen seed and the prefrontal cortex. The seed was selected based on voxels in the amygdala that showed decreased activation from pre- to post-intervention (see Results) in both groups. These voxels were mapped back into each individual and used to average the functional time series. This waveform was then correlated with all other voxels in the bilateral prefrontal cortex (global signal, white matter signal, ventricle-CSF signal and motion parameters were included as nuisance variables). The map of regression coefficients was then used for higher-level group analysis. For post-hoc analyses and the analysis of correlations with PSS and BAI scores, values for clusters that were extracted were reported below and Spearman's ρ was calculated in SPSS (SPSS, 2004). P-values for all correlations are reported uncorrected.

## 3 Results

### 3.1 Baseline analysis of affect labeling in healthy participants

**Fig. 1:**
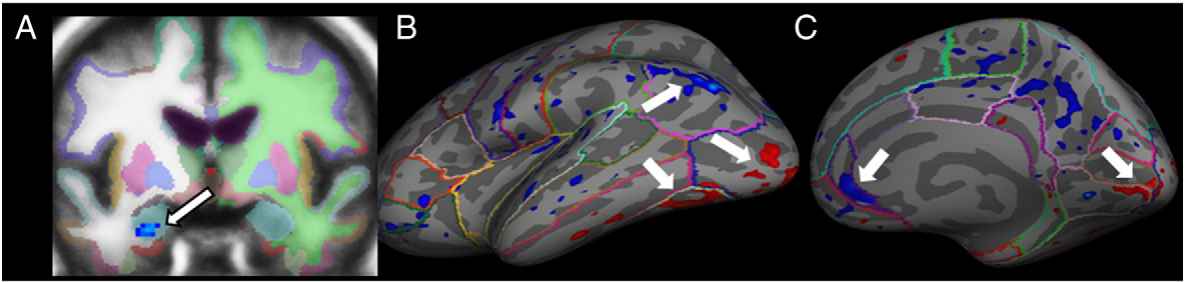
When labeling the affect, compared to the gender of facial expressions, healthy participants (N = 26) show decreased activation in the left amygdala (A; p = 0.0121, multiple comparison corrections within area of bilateral amygdalae), the right rostral ACC (C; p = 0.0314; multiple comparison corrections within mask of the frontal cortex/insula), and left inferior parietal cortex (B; p = 0.0321, multiple comparison corrections for entire brain for this and all following clusters) and increased activation in the left lateral occipital (B; p = 0.0087), left fusiform (B; p = 0.0099), and right lingual cortex (C; p = 0.0105).

Healthy participants were first investigated during affect labeling compared to gender labeling of facial expressions (angry, happy, and neutral combined). In line with previous research, the contrast affect vs. gender labeling yielded decreased activation in the amygdala region of interest (see Fig. 1, Table 2). Within the region of interest in the frontal and insular cortex, we found only decreased activation in the right rostral ACC, but no increased activation in VLPFC regions. Furthermore, exploratory whole brain analysis showed decreased activation in the left inferior parietal cortex and increased activation in the left lateral occipital, left fusiform, and right lingual cortex.

### 3.2 Baseline comparisons between GAD and healthy participants

Pre-treatment, PSS scores of GAD patients (M:22.92, SD:5.26) were significantly higher than those of healthy participants (M:14.60, SD:4.73; independent sample *t*-test: *t*(49) = − 5.94, p < .001), indicating that patients felt more stressed than controls.

**Fig. 2:**
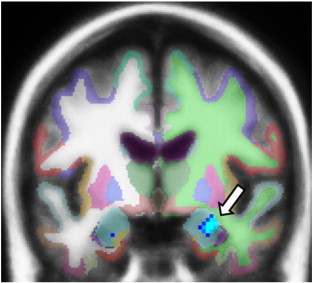
GAD patients (N = 26) show greater activation in a cluster in the right amygdala when viewing neutral facial expressions when compared to healthy participants (N = 26; p = 0.0001; size = 440 mm3; multiple comparison corrections within area of bilateral amygdalae; cluster overlaid over a FreeSurfer subcortical parcellation image).

The group comparison of amygdala activation between GAD patients and healthy participants at baseline revealed no significant difference in response to angry faces. However, in response to neutral faces, GAD patients showed a significantly greater activation in a cluster in the right amygdala (p = 0.0001; size mm^3^ = 440.0, MNI coordinates x,y,z:26,− 9,− 21; Z = − 2.81; Fig. 2). There were no significant correlations between the extracted signal from this cluster and values on the BAI or PSS scales. To test the specificity of the findings of enhanced amygdala activation in response to neutral facial expressions, we additionally investigated the group difference in response to happy faces. There was no significant difference between GAD patients and healthy participants (largest cluster in the left amygdala: p = 0.13; Z = − 2.11). No further clusters were identified to show a group difference in response to the angry, neutral, or happy facial expressions across the rest of the brain.

### 3.3 Pre–post intervention changes in reported symptoms and brain activation in GAD patients

ANOVAs were conducted to assess pre–post changes in brain activation and reported stress and anxiety across both interventions (ANOVA main effect) and to test whether changes were greater in the MBSR than the SME group (ANOVA group-by-time interaction).

Regarding PSS score, there was a significant main effect of time (F(1,24) = 30.32, p < .001), but no significant group-by-time interaction (F(1,24) = .013, p = .91), indicating that both groups showed an improvement of perceived stress scores, and that the MBSR group did not show a stronger decrease than the SME group. Results regarding symptom improvements measured with the BAI have been reported elsewhere (Hoge et al., in press). Specifically, Hoge et al. found that MBSR participants showed a significantly greater decrease in BAI scores than SME participants.

#### 3.3.1 Amygdala activation

Investigation of amygdala activation revealed a highly significant pre–post intervention decrease in activation in a cluster in the right amygdala for the collapsed GAD sample in response to neutral facial expressions (p = 0.0003, size mm^3^:784, MNI coordinates x,y,z:30,− 5,− 19; Z = − 3.77). However, the pre–post change was not significantly different in the two treatment intervention groups. There was also no difference between the two intervention groups at baseline. There was no significant main effect or group-by-time interaction in decreases in amygdala response to the angry facial expressions. A decrease in response to the happy facial expressions in the MBSR group after the intervention in a cluster in the left amygdala missed significance (p = 0.054, size mm3:176, MNI coordinates x,y,z:− 30,− 3,− 27; Z = − 2.14), and the group-by-time interaction was not significant. Activation changes were not correlated with changes in questionnaire scores.

#### 3.3.2 Frontal and insular activation

Regarding the frontal and insular cortex, there were no significant changes in response to the angry facial expressions for the collapsed GAD sample. In response to neutral facial expressions, there were clusters in the right caudal middle frontal (p = 0.009, size mm^2^:473, MNI coordinates x,y,z:34,8,34, Z = − 3.61) and the right lateral orbitofrontal cortex (p = 0.0054, size mm^2^:380, MNI coordinates x,y,z:15,30,− 25, Z = − 3.49), where participants showed pre–post activity decreases.

**Fig. 3:**
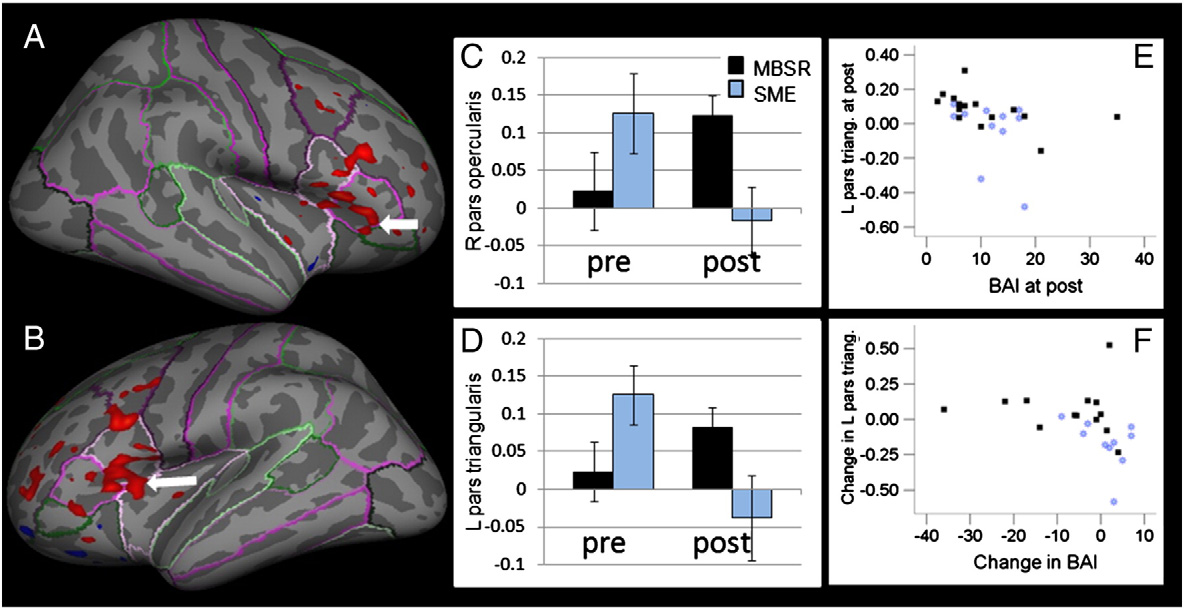
MBSR participants (N = 15) show stronger increases in brain activation in clusters in the right pars opercularis (A; p = 0.0156; multiple comparison corrections within mask of the frontal cortex/insula) and left pars triangularis (B; p = 0.0015), i.e., ventrolateral prefrontal regions when viewing neutral emotional expressions than SME participants (N = 11). Extracted averaged values from the clusters in the right pars opercularis (C), and the left pars triangularis (D) for the MBSR (black) and SME (blue) groups when viewing neutral facial expressions at pre- and post-interventions (error bars indicate standard errors). Signal in the cluster in the left pars triangularis is correlated with scores on the Beck Anxiety Inventory (BAI) at post-intervention (E; ρ = − .645, p < .001, uncorrected) and the pre–post intervention change in this cluster is correlated with the change in BAI (F; ρ = − .617, p = .001, uncorrected).

The MBSR and SME groups showed differential pre–post changes in frontal cortical activation in response to both neutral and angry facial expressions. Specifically, in response to neutral faces MBSR participants showed a stronger increase than SME patients in VLPFC regions, namely in the right pars opercularis and left pars triangularis (Table 3, Fig. 3). Post hoc tests on the extracted signal averaged over the cluster showed that in the right pars opercularis, there was no significant difference between the treatment group pre-interventions (*t*(24) = 1.37, p = .184), a greater activation in the MBSR than the SME group at post-intervention (*t*(16.95) = − 2.65, p = .017), and a marginally significant increase in the MBSR group (*t*(14) = − 2.05, p = .060). In the left pars triangularis, there was no significant difference between the SME and MBSR groups at pre-intervention (*t*(24) = 1.77, p = .090). The difference between the activation in the MBSR and the SME group at post-intervention missed significance (*t*(14.25) = − 1.92, p = .075), and there was no significant pre- to post-increase in the MBSR group (*t*(14) = − 1.40, p = .183).

We then assessed whether the brain activation in the reported clusters was correlated with scores on the PSS and BAI. There was a strong negative correlation between post-intervention BAI scores and post-intervention activation in the cluster in the left pars triangularis (ρ = − .645, p < .001; Fig. 3E). Higher activation in this cluster was associated with lower anxiety symptoms. Furthermore, there was a significant correlation between the pre–post activation change in this cluster and the change in the BAI (ρ = − .617, p = .001), such that decreases in anxiety symptoms over time were related to increases in brain activation in the cluster in the left VLPFC. Correlations between BAI symptoms and values in the right pars opercularis, and correlations with the PSS were not significant.

**Fig. 4:**
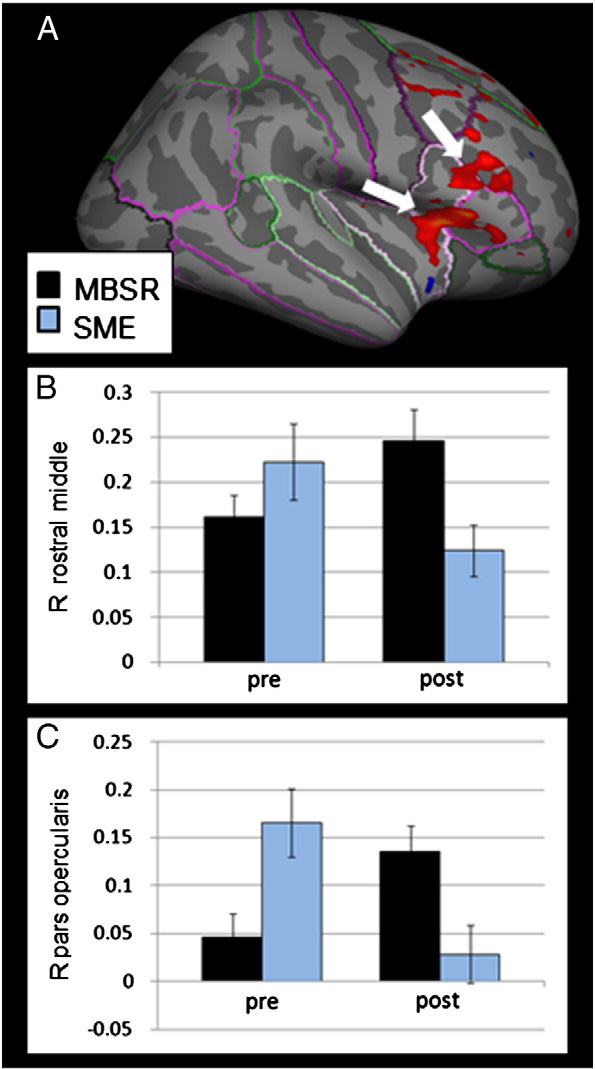
MBSR participants (N = 15) show stronger pre–post increases than SME participants (N = 11) in two clusters in the right VLPFC when viewing angry facial expressions (A; pars opercularis: p = 0.0003; rostral middle frontal gyrus: p = 0.0018; multiple comparison corrections within mask of the frontal cortex/insula). Extracted averaged signal for the MBSR (black) and SME (blue) groups at pre- and post-interventions in the right rostral middle frontal gyrus, reaching into the pars opercularis (B) and right pars opercularis, reaching into the pars triangularis and insula (C; error bars indicate standard errors).

Further significant group-by-time interactions were found in response to angry faces. MBSR participants showed stronger increases in activation than SME participants following the intervention in the right pars opercularis/triangularis, reaching into the insula as well as in the right rostral middle frontal cortex reaching into the pars opercularis (Table 3, Fig. 4). Post hoc tests conducted on the extracted averaged signal showed that in the right rostral middle frontal cortex, there was no significant difference between the groups at pre-intervention (*t*(16.33) = 1.25, p = .228), but MBSR participants showed significantly greater activation than SME participants at post-intervention (*t*(24) = − 2.50, p = .020). The increase in the MBSR group was significant (*t*(14) = − 2.29, p = .038). In the right pars opercularis, SME participants showed greater activation at pre-intervention than MBSR participants (*t*(24) = 2.88, p = .008), despite the randomization. However, MBSR participants showed a significantly greater activation in this cluster than SME participants at post-intervention (*t*(24) = − 2.67, p = .014), and the increase in the MBSR group was significant (*t*(14) = − 3.06, p = .009). Neither the post-intervention signal values nor pre–post changes were correlated with scores on the BAI or PSS. Additional results from the exploratory whole brain analysis are reported in Table 4.

### 3.4 Changes in amygdala–prefrontal functional connectivity

The cluster in the right amygdala where a decrease in BOLD response was found pre- to post-intervention in the entire GAD sample was used as a seed for a functional connectivity analysis, to assess whether this change was related to differential coupling with prefrontal brain regions in the two groups. The correlation between the time-course of this seed region in the first affect labeling block and the time-course in the rest of the brain was calculated.

When comparing the connectivity between GAD patients and healthy controls pre-intervention, there were no areas across the brain that showed a significant difference in functional connectivity with the amygdala seed region.

**Fig. 5:**
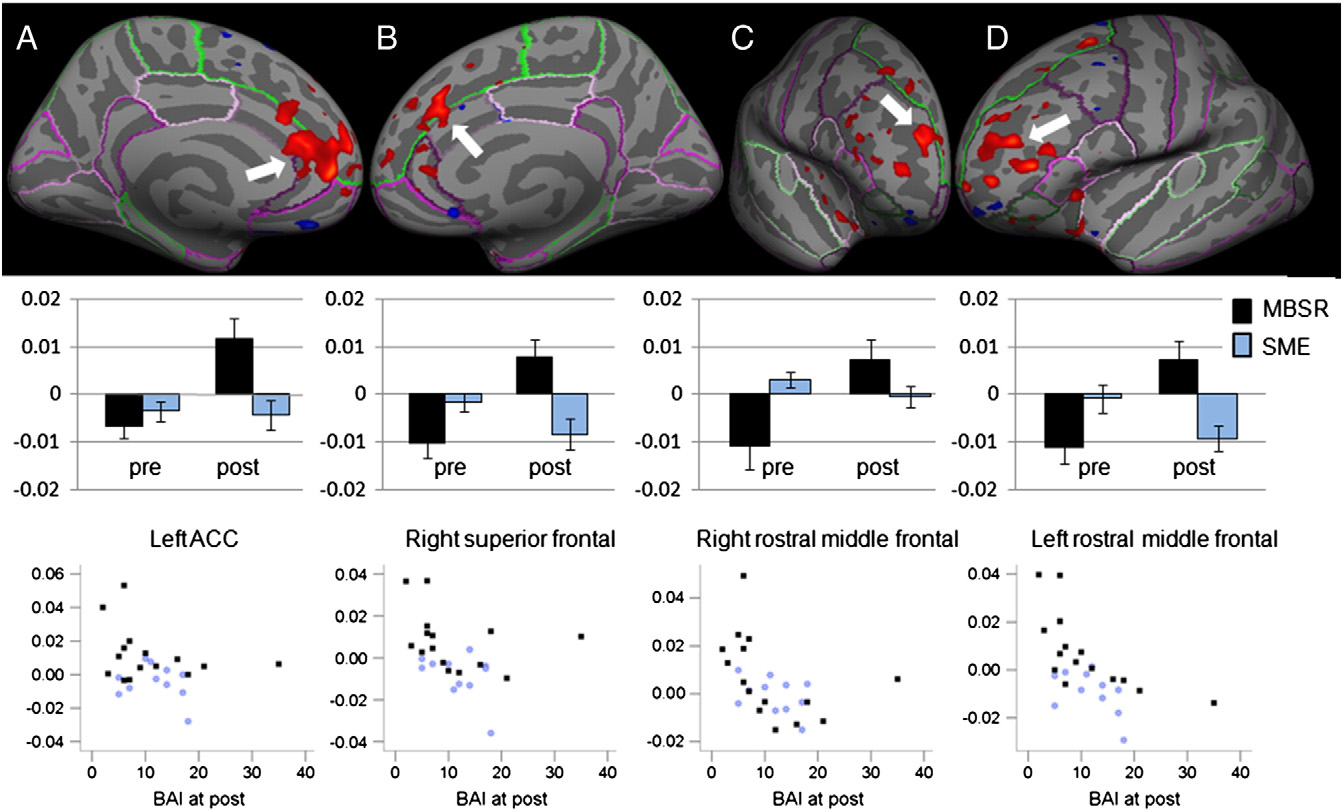
Functional connectivity between the seed region in the right amygdala and several regions in the frontal cortex increased from pre- to post-intervention in GAD patients who underwent the MBSR program (N = 15), but not in those who underwent the SME class (N = 11). Anatomical location displayed on an inflated surface with FreeSurfer cortex parcellations (top row), regression coefficients extracted from the clusters from the MBSR (black) and SME (blue) participants at pre- and post-interventions (middle row) and scatter plots of regression coefficients (y-axis) and Beck Anxiety Inventory (BAI, x-axis) for MBSR and SME participants at post (bottom row) for the left rostral anterior cingulate cortex (ACC, column A, pre- to post increase in connectivity: p = 0.0002, multiple comparison corrections within mask of the frontal cortex/insula; correlation with BAI scores: ρ = − .229, ns, uncorrected), right superior frontal cortex (column B, pre–post increase: p = 0.04; correlation: ρ = − .470, p = .015), right rostral middle frontal cortex (column C, pre–post increase: p = 0.03; correlation: ρ = − .572, p = .002), and left rostral middle frontal cortex (column D, pre–post increase: p = 0.01; correlation: ρ = − .646, p < .001).

We then examined changes in the functional connectivity in GAD patients who underwent the MBSR program, and found a significant increase in functional connectivity of this seed with several areas in the frontal cortex: the left rostral anterior cingulate cortex (ACC), the left rostral middle frontal cortex, the right rostral middle frontal cortex, and the right superior frontal cortex (Table 5, Fig. 5). The connectivity changed from a negative to a positive correlation at post-intervention in all clusters.

In the SME group, there were no regions that showed a change in connectivity with the seed in the right amygdala. When investigating the group-by-time interaction on the surface and applying multiple comparison corrections across the entire search space, increases in functional connectivity between the seed and the left rostral middle frontal cortex (p = 0.0002) and the right superior frontal cortex (p = 0.004) were significantly greater in the MBSR than the SME group. The region in the left rostral ACC and right rostral middle frontal cortex missed significance.

To assess the potential clinical relevance of these connectivity measures, we then examined their correlations with the BAI (Fig. 5). Post-intervention scores on the BAI were negatively correlated with the connectivity of the amygdala to: the clusters in the left rostral middle frontal cortex (ρ = − .646, p < .001), the right rostral middle frontal cortex (ρ = − .572, p = .002), and the right superior frontal cortex (ρ = − .470, p = .015). Higher positive connectivity between the signal in the amygdala seed region and the signal in these clusters was associated with lower anxiety symptoms. Furthermore, the pre–post change in BAI scores was correlated with the pre–post change of functional connectivity of the right amygdala seed region and the left rostral middle frontal cortex (ρ = − .648, p < .001), the right rostral middle frontal cortex (ρ = − .487, p = .018) and the right superior frontal cortex (ρ = − .424, p = .044).

## 4 Discussion

This study reveals altered neural responses to neutral facial expressions in GAD patients compared to controls at baseline. The data also revealed neural correlates underlying the beneficial effects of an 8-week MBSR intervention on clinical symptoms in GAD patients as compared to an active control intervention.

### 4.1 Baseline analysis of affect labeling in healthy participants

To establish comparability of healthy participants' brain activation during affect labeling with previous findings, we first assessed baseline measures of affect vs. gender labeling. In line with previous research (Foland et al., 2008; Hariri et al., 2000; Lieberman et al., 2007), and in line with the assumption that naming the affect of emotional stimuli reduces arousal, significantly reduced amygdala activation was found during affect vs. gender labeling. Furthermore, subjects showed greater activation in brain areas involved in visual processing, such as the lateral occipital, fusiform and lingual gyri, known for their functions in object recognition and face perception (Grill-Spector et al., 2004, 2011; Kim et al., 1999). The stronger visual cortex activity during affect labeling might be related to higher attentional engagement and/or greater motivational relevance (Bradley et al., 2003). Along the same line, brain activation was lower in brain regions involved in task-unrelated cognitions or mind-wandering (Buckner et al., 2008) during affect compared to gender labeling. The current study did not confirm increased VLPFC activation during affect compared to gender labeling.

### 4.2 Baseline comparison between GAD and healthy participants

While it has been repeatedly documented that several anxiety disorders are associated with amygdala hyperactivation (Breiter and Rauch, 1996; Etkin and Wager, 2007; Evans et al., 2008; Shin et al., 2005), the literature specifically pertaining to GAD has been more variable (Etkin, 2011). Studies in adults with GAD using consciously presented emotional facial expressions have mostly not found amygdala hyperactivation (Monk et al., 2006; Palm et al., 2011; Whalen et al., 2008), and one study found hypoactivation (Blair et al., 2008). The present study also found no stronger amygdala response to emotional faces (angry and happy facial expressions), but did reveal that GAD patients showed stronger amygdala activation in response to neutral facial expressions compared to healthy participants. In congruence with our findings, higher amygdala activation in response to neutral facial expressions has also been found in subjects with higher levels of state anxiety (Somerville et al., 2004), as well as in subjects with anxious and fearful childhood temperaments (Blackford et al., 2010; Schwartz et al., 2003a, 2003b). Furthermore, exaggerated amygdala response has been found to nonconsciously presented emotional facial expressions (Monk et al., 2008) and when anticipating viewing neutral or aversive pictures (Nitschke et al., 2009) in GAD. Together, these findings suggest that it might be stimuli with greater ambiguity that cause greater amygdala activation in GAD patients (see (Whalen, 1998)). This observation lends evidence to the ‘intolerance of uncertainty’ model of GAD, which posits that patients are particularly uncomfortable with stimuli in which the meaning is unclear (Dugas et al., 1998). It is plausible that uncertainty itself is more disturbing than the angry faces, which can more easily be identified and categorized. Interestingly, research using a variety of protocol tasks has found that GAD patients interpret ambiguous stimuli as more threatening than non-anxious controls; for example, ambiguous homophones were identified with a more threatening meaning (Mathews et al., 1989), and ambiguous sentences were seen as more threatening by GAD patients than controls (Eysenck et al., 1991). Thus, not only might neutral faces evoke a greater response due to intolerance of uncertainty, but also because they might be classified by GAD patients as actually threatening. It is also possible that negative stimuli might initiate a compensatory state, consistent with the theory by Borkovec (Borkovec, 1994; Borkovec et al., 2004), thereby diminishing arousal.

### 4.3 Changes in brain activation from pre- to post-intervention

Following both interventions, activations in the right amygdala in response to the neutral facial expressions were reduced. Possibly, brain activation might have decreased because both interventions were effective in reducing stress, documented by a significant main effect of time on PSS scores, though no group-by-time interaction on this measure. The absence of the superiority of MBSR in reducing stress over an active control intervention has previously been documented in regard to a similar control intervention, the Health Enhancement Program (MacCoon et al., 2008). However, due to the absence of a no-treatment control group, we cannot exclude the possibility that amygdala activation might have been reduced in both groups due to habituation of novelty in the current study (arising from the presentation of identical stimuli at both pre- and post-interventions). Desbordes et al. (2012) have recently reported that mindfulness training leads to decreased amygdala activation in response to emotional pictures, while no such effect was observed in a control intervention, demonstrating that mindfulness can have effects of reducing amygdala activity beyond habituation effects.

While the generic stress management education program was similarly effective in reducing perceived stress, participation in MBSR led to superior GAD symptom improvement compared to SME (see (Hoge et al., in press) for a detailed presentation of the results). Furthermore, MBSR participants showed greater increases in brain activity in areas within the VLPFC than SME participants. Activation in a cluster in the left VLPFC was negatively correlated with clinical anxiety scores at post, and the pre–post change in brain activation correlated negatively with pre–post changes in the BAI, suggesting the clinical relevance of these changes.

These findings are interesting in the light of the emotion dysregulation model of GAD (Mennin et al., 2005), which describes that individuals with GAD have difficulty identifying and describing emotions. Mindfulness practice aims at enhancing the awareness of present moment experiences, including emotions (Kabat-Zinn, 1990), thereby facilitating the identification and description of emotions. In the context of a task that employs the labeling of affective facial expressions, which is known to recruit activation of the VLPFC (Lieberman et al., 2007), we demonstrated that MBSR had an advantageous effect over SME in the recruitment of VLPFC activation. One might therefore speculate that MBSR enhances the labeling of emotions, reflected in enhanced VLPFC recruitment, which leads to beneficial effects on symptom reduction in GAD patients, thereby helping to address this population's deficits.

Preliminary findings by Maslowsky et al. (2010) suggest that CBT and treatment with SSRIs also lead to increased VLPFC activation in response to emotional facial expressions in GAD patients, indicating that increased VLPFC activation might not be mindfulness specific, but might more generally represent enhanced emotion regulation capacities. That study had several methodological limitations though, such as a small sample size (n = 7 per group), no multiple comparison corrections, and the absence of a control condition. The current study establishes that enhanced VLPFC activation follows from successful treatment and not as a result of repeated stimuli presentation, and that it corresponds to reported symptom improvements. However, research is needed to determine whether enhanced VLPFC activation represents a causal mechanism or an effect of symptom improvement.

### 4.4 Changes in amygdala–prefrontal functional connectivity

We found increases in connectivity between the amygdala and several regions of the prefrontal cortex after MBSR but not after the SME course. To the best of our knowledge, this is the first study to report modified amygdala–prefrontal connectivity following treatment in GAD. Prefrontal regions included bilateral dorsolateral and bilateral dorsomedial prefrontal regions and the dorsal ACC. The strength of the coupling between these regions was negatively correlated with anxiety symptom severity, assessed with the BAI, at post intervention. The pre–post change in coupling was also negatively correlated with pre–post changes in the BAI, indicating the clinical relevance of the strengthened connectivity.

Before the interventions, we did not detect differences in connectivity between the GAD patients and the healthy participants. Conversely, Etkin et al. (2009) reported lower connectivity of the amygdala with several brain areas in GAD patients as compared to controls, including the dorsal/midcingulate, VLPFC, and insula. Based on findings that activation in the dorsal ACC tracks with sympathetic nervous system arousal (Critchley, 2005), Etkin et al. speculate that the decreased coupling of the amygdala to this network might be related to abnormalities in modulation of the autonomic nervous system seen in GAD. The enhanced amygdala–dorsal ACC coupling that we found following the mindfulness-based program might be related to improved regulation of arousal states. This is aligned with the finding that meditation practice positively influences regulation of the autonomic nervous system (Tang et al., 2009).

Increased connectivity was also observed between the amygdala and the dorsolateral PFC during the affect label task following MBSR. Etkin et al. (2009) found stronger amygdala – dorsolateral PFC coupling in GAD patients as compared to healthy controls in the resting state, which was negatively correlated with BAI scores – thus, higher connectivity was indicative of lower anxiety. Those authors speculate that this coupling may reflect an engagement of a compensatory executive control system to regulate excessive anxiety (Etkin et al., 2009).^4^ We found an increase over time in coupling between these regions during the task following the MBSR intervention, which was also negatively correlated with BAI scores. These findings suggest modified emotion regulation following mindfulness training in GAD patients. A typical, and mostly ineffective strategy employed by GAD patients to cope with or avoid the experience of emotions is worrying (Borkovec, 1994; Borkovec et al., 2004; Mennin et al., 2002). We postulate that patients in this study have learned a different, more adaptive strategy to regulate emotions, thereby helping to circumvent maladaptive emotion regulation strategy use (Mennin et al., 2002).

Surprisingly, MBSR participants showed a change from a negative connectivity between the frontal regions and the amygdala (i.e., an anti-correlation) pre-intervention to a positive connectivity (i.e., positive correlation between the amygdala and prefrontal activations). In the context of emotion regulation, prefrontal regions are thought to down-regulate limbic reactivity, yielding a negative connectivity (Banks et al., 2007; Etkin et al., 2006; Lee et al., 2012). Given that mindfulness involves observing physical and emotional responses with a detached and accepting attitude, as opposed to down-regulation or suppression, we speculate that the observed positive connectivity might be related to an engagement in active monitoring of arousal rather than an attempt to down-regulate the emotional response. Such a pattern would be in strong contrast to the avoidance of internal experiences that is a salient feature of GAD, as described by several current theoretical models (see (Behar et al., 2009)). Mindful engagement with thoughts and emotions has been referred to as ‘decentering’ (Fresco et al., 2007) — the capacity to observe these phenomena as temporary, objective events in the mind, rather than reflections of the self that are necessarily true. Future studies could experimentally manipulate decentering – e.g., instructing participants to relate to experiences from different internal perspectives – to test whether the change in functional connectivity observed here relates to this altered perception of emotions.

A limitation of this study is its small sample size. At baseline, the two GAD intervention groups showed differences in the magnitude of activation in VLPFC clusters, despite randomization. This difference was significant in one cluster, the right pars opercularis in response to angry expressions (but not in the other three clusters). We therefore can't exclude the possibility that baseline group differences contributed to the interaction effect in this cluster. When performing additional post-hoc analyses after excluding participants with co-morbid SAD, major depressive disorder and medicated subjects, findings proved to remain relatively invariant (see Supplementary materials), indicating robustness of the results. Nevertheless, future studies need to replicate these findings with bigger samples.

A limitation of this study is its small sample size. At baseline, the two GAD intervention groups showed differences in the magnitude of activation in VLPFC clusters, despite randomization. This difference was significant in one cluster, the right pars opercularis in response to angry expressions (but not in the other three clusters). We therefore can't exclude the possibility that baseline group differences contributed to the interaction effect in this cluster. When performing additional post-hoc analyses after excluding participants with co-morbid SAD, major depressive disorder and medicated subjects, findings proved to remain relatively invariant (see Supplementary materials), indicating robustness of the results. Nevertheless, future studies need to replicate these findings with bigger samples.

To conclude, our findings demonstrate that mindfulness training is associated with enhanced activation in and connectivity between several brain regions that are known to be crucial to successful emotion regulation, both for healthy and anxiety disorder populations (Kim et al., 2011a). The study elucidates the neural correlates of symptom improvements following treatment with MBSR, indicating that this medication-free and cost-effective group intervention may influence brain activation and functional connectivity in a direction with important relevance for mental health.

The following are the Supplementary data related to this article.

Supplementary materials Table 1 Post-hoc analyses of all reported findings when excluding patients with comorbid social anxiety disorder (SAD), comorbid major depressive disorder (MDD), and medicated subjects. Fields where the significance of the findings changed are bolded.

Supplementary data to this article can be found online at http://dx.doi.org/10.1016/j.nicl.2013.03.011.

## Figures and Tables

**Table 1:** Age, gender, and education of generalized anxiety disorder (GAD) patients and healthy participants, as well as separately for the two GAD subgroups (MBSR and SME participants).

	GAD	Healthy	Test	GAD MBSR	GAD SME	Test
Age mean years (SD)	37.9 (12.2)	35.7 (9.3)	Independent sample *t*-test *t*(50) = .76, p = .45	38.5 (13.3)	35.6 (10.8)	Independent sample *t*-test *t*(26) = − .58, p = .57
Gender	14 females, 12 males	16 females, 10 males	*χ*^2^(Fisher's exact test) p = .78	9 females, 6 males	5 females, 6 males	*χ*^2^ = .54, asymp significance = .46
Education mean years (SD)	17.5 (2.5)	16.9 (1.9)	Independent sample *t*-test *t*(46.28) = 1.05, p = .30	17.1 (2.8)	18.2 (2.1)	Independent sample *t*-test *t*(24) = 1.11, p = .28

**Table 2:** Brain activation in healthy participants during affect labeling (compared to gender labeling) of emotional facial expressions (angry, happy, and neutral faces combined).

Brain region of maximum	Cluster-wise p	Size (mm^2^/mm^3^)	Max (Z)	MNI-x	MNI-y	MNI-z
*Amygdala region of interest*						
Left amygdala	0.0121	248	− 2.64	− 26	− 3	− 27

*Prefrontal/insular region of interest*						
Right rostral ACC	0.0314	251	− 2.98	5	31	− 2

*Exploratory whole brain analysis*						
Left inferior parietal	0.0321	480	− 4.34	− 40	− 75	33
Left lateral occipital	0.0087	582	3.47	− 17	− 99	− 9
Left fusiform	0.0099	564	3.40	− 38	− 68	− 17
Right lingual	0.0105	463	4.00	14	− 93	− 9

**Table 3:** Brain regions within the prefrontal/insula regions of interest where MBSR participants showed stronger pre–post intervention increases than SME participants when viewing neutral and when viewing angry facial expressions.

Brain region of maximum	Cluster-wise p	Size (mm^2^)	Max (Z)	MNI-x	MNI-y	MNI-z
*Neutral facial expressions*						
Right pars opercularis	0.0156	339	3.09	46	20	9
Left pars triangularis	0.0015	470	2.91	− 34	26	9

*Angry facial expressions*						
Right pars opercularis	0.0003	583	3.52	34.6	21.7	11.7
Right rostral middle frontal	0.0018	473	2.93	45.0	28.5	23.9

**Table 4:** Areas of significant change in BOLD signal in response to angry and neutral facial expressions in the MBSR and the SME groups in exploratory whole brain analyses.

Brain region of maximum	Cluster-wise p	Size (mm^2^)	Max (Z)	MNI-x	MNI-y	MNI-z
*MBSR group*						
*Neutral facial expressions*						
Right fusiform gyrus	0.01671	537	− 3.26	34	− 45	− 19
*Angry facial expressions*						
Right precuneus	0.03058	498	3.19	9	− 49	45
Left lateral occipital	0.02440	496	− 3.96	− 43	− 75	− 10
						
*SME group*						
*Neutral facial expressions*						
Right superior temporal	0.02115	431	− 3.83	55	− 31	2
Left banks superior temporal sulcus	0.00030	1010	− 3.71	− 57	− 46	− 2
Left pars opercularis	0.00060	657	− 3.20	− 36	10	24
Left pars opercularis	0.00927	462	− 3.76	− 50	19	12
*Angry facial expressions*	No significant clusters					

**Table 5:** Areas of increased connectivity with the cluster in the right amygdala in the GAD group that underwent the MBSR program.

Brain region of maximum	Cluster-wise p	Size (mm^2^)	Max (Z)	MNI-x	MNI-y	MNI-z
RH rostral middle frontal	0.03430	274	3.13	24	51	19
RH superior frontal	0.04371	264	3.12	10	28	36
LH rostral anterior cingulate	0.00020	692	3.46	− 11	44	5
LH rostral middle frontal	0.01137	319	3.23	− 33	45	18

## Footnotes

2 For a review of findings with pediatric GAD, see 27 Strawn et al. (2012): Establishing the neurobiologic basis of treatment in children and adolescents with generalized anxiety disorder. *Depress Anxiety*. 29:328–339.

3 Additional post-hoc analyses were performed excluding patients with comorbid SAD, excluding patients with comorbid major depressive disorder and excluding medicated subjects. Full results are reported in the Supplementary materials. The major conclusions of the data analyses remained the same when excluding these subgroups. We have opted to report results based on the full sample here, since comorbid conditions in GAD are the norm.

4 The dorsolateral PFC receives minimal direct projections from the amygdala and has only weak projections to it, so projections might be relayed through other prefrontal areas, such as the dorsal ACC 77. Ray and Zald (2012): Anatomical insights into the interaction of emotion and cognition in the prefrontal cortex. *Neurosci Biobehav Rev*. 36:479–501.
