# Supplementary material for: Neural mechanisms of symptom improvements in generalized anxiety disorder following mindfulness training
Source: Neuroimage Clin. 2013 Mar 25;2:448–58. doi: 10.1016/j.nicl.2013.03.011 (PMC3777795; doi:10.1016/j.nicl.2013.03.011)
Supplement: Supplementary materials Table 1 — Post-hoc analyses of all reported findings when excluding patients with comorbid social anxiety disorder (SAD), comorbid major depressive disorder (MDD), and medicated subjects. Fields where the significance of the findings changed are bolded. [file mmc1.doc]

Table 1, Supplementary materials: Post-hoc analyses of all reported findings when excluding patients with comorbid social anxiety disorder (SAD), comorbid major depressive disorder (MDD), and medicated subjects. Fields where the significance of the findings changed are bolded.

|  | Full sample (MBSR: N=15, SME: N=11, healthy: N=26) | SAD excluded (MBSR: N=10, SME: N=10, healthy: N=26) | MDD excluded (MBSR: N=12, SME: N=10, healthy: N=26) | Medication excluded (MBSR: N=12, SME: N=10, healthy: N=26) |
| --- | --- | --- | --- | --- |
| **Baseline comparison between GAD and healthy participants** |  |  |  |  |
| PSS scores,  GAD vs. healthy | t=-5.94,  p <.001 | t(41)=-5.24, p<.001 | t(44)=-4.95, p<.001 | t(45)=5.50, p<.001 |
| amygdala, GAD vs. healthy, neutral expressions | t(50)=-3.21, p=.002 | t(44)=-2.86, p=.007 | t(46)=-2.90, p=.006 | t(46)=-3.11, p=.003 |
| Correlation PSS with amygdala, angry expressions, GAD group | ρ=.50,  p=.01 | ρ=.467,  p=.038 | ρ=.61,  p=.003 | ρ=.43,  p=.046 |
| **Pre-post change in GAD patients** |  |  |  |  |
| PSS score, collapsed sample, main effect of time | F(1,24)=30.32, p<.001 | F(1,18)=27.72, p<.001 | F (1,20)=25.17, p<.001 | F(1,20)=22.38, p<.001 |
| PSS score, group by time interaction | F(1,24)=.01, p=.91 | F(1,18)=.33,  p=.57 | F(1,20)=.33,  p=.57 | F(1,20)=.11,  p=.74 |
| Amygdala activation, neutral faces, collapsed sample, main effect of time | F(1,24)=12.32,  p=.002 | F(1,18)=11.39,  p=.003 | F(1,20)=8.15,  p=.010 | F(1,20)=9.94,  p=.005 |
| Amygdala activation, group by time interaction | F(1,24)=.69,  p=.42 | F(1,18)=2.76;  p=.11 | F(1,20)=1.15,  p=.30 | F(1,20)=.30,  p=.59 |
| Right pars opercularis, neutral expressions, group by time interaction | F(1,24)=12.19, p=.002 | F(1,18)=8.61,  p=.009 | F(1,20)=10.54,  p=.004 | F(1,20)=18.08,  p<.001 |
| Right pars opercularis, neutral expressions, pre difference between MBSR and SME | t(24)=1.37, p=.18 | t(18)=1.24,  p=.23 | t(20)=1.80, p=.09 | t(11.044)=.62,  p=.55 |
| Right pars opercularis, neutral expressions, pre-post increase in MBSR | t(14)=-2.05, p=.06 | t(9)=-1.77,  p=.11 | t(11)=-1.80,  p=.10 | **t(11)=-2.52,  p=.03** |
| Left pars triangularis, neutral expressions, group by time interaction | F(1,24)=11.83,  p=.002 | F(1,18)=12.14,  p=.003 | F(1,20)=7.49,  p=.013 | F(1,20)=11.60,  p=.003 |
| Left pars triangularis, neutral expressions, pre difference between MBSR and SME | t(24)=1.77, p=.09 | t(18)=1.254, p=.23 | **t(20)=2.704,  p=.01** | t(20)=1.457,  p=.16 |
| Left pars triangularis, neutral pre-post increase in MBSR | t(14)=-1.40, p=.18 | t(9)=-1.69,  p=.13 | t(11)=-1.03,  p=.32 | t(11)=-.272,  p=.79 |
| BAI – left pars triangularis correlation at post | ρ=-.65,  p<.001 | ρ=-.64,  p=.003 | ρ=-.61,  p=.003 | ρ=-.64,  p=.001 |
| BAI change – change in left pars triangularis correlation | ρ=-.62,  p=.002 | ρ=-.57,  p=.012 | ρ=-.53,  p=.021 | ρ=-.70,  p=.001 |
| Right pars opercularis, angry faces, group by time interaction | F(1,24)=18.98,  p<.001 | F(1,18)=13.05,  p=.002 | F(1,20)=16.52,  p=.001 | F(1,20)=16.68,  p=.001 |
| Right pars opercularis, angry faces, pre difference MBSR vs. SME | t(24)=2.88, p=.008 | t(18)=2.66,  p=.016 | t(20)=2.40,  p=.026 | t(20)=2.41,  p=.026 |
| Right pars opercularis, angry faces, pre-post increase in MBSR | t(14)=-3.06, p=.009 | t(9)=-2.61,  p=.028 | t(11)=-3.07,  p=.011 | **t(11)=-2.18,  p=.052** |
| Right rostral middle frontal cortex, angry faces, group by time interaction | F(1,24)=12.69,  p=.002 | F(1,18)=23.29,  p<.001 | F(1,20)=8.22,  p=.010 | F(1,20)=10.35,  p=.004 |
| Right rostral middle frontal cortex, angry faces, pre difference between MBSR and SME | t(16.33)=1.25, p=.23 | t(14.573)=1.68,  p=.12 | t(20)=.87,  p=.40 | t(13.196)=.75,  p=.47 |
| Right rostral middle frontal cortex, angry faces, pre-post increase in MBSR | t(14)=-2.29, p=.038 | t(9)=-3.60,  p=.006 | **t(11)=-1.83,  p=.094** | **t(11)=-1.61,  p=.135** |
| **Amygdalar prefrontal functional connectivity** |  |  |  |  |
| Left rACC, pre-post change in MBSR group | t(14)=-4.43,  p=.001 | t(9)=-3.35,  p=.008 | t(11)=-3.94,  p=.002 | t(11)=-4.06,  p=.002 |
| Left rACC, group by time interaction | F(1,24)=10.79,  p=.003 | F(1,18)=13.60,  p=.002 | F(1,20)=8.29,  p=.009 | F(1,20)=9.19,  p=.007 |
| Left rACC, BAI – functional connectivity correlation at post | ρ=-.23,  p=.26 | ρ=-.24,  p=.32 | ρ=-.24,  p=.28 | ρ=-.33,  p=.13 |
| Left rACC, BAI change – change in functional connectivity correlation | ρ=-.26,  p=.24 | ρ=-.38,  p=.11 | ρ=-.16,  p=.53 | ρ=-.31,  p=.20 |
| Left rostral middle frontal cortex, pre-post change in MBSR group | t(14)=-3.70,  p=.002 | t(9)=-2.83,  p=.020 | t(11)=-2.90,  p=.014 | t(11)=-3.40,  p=.006 |
| Left rostral middle frontal cortex, group by time interaction | F(1,24)=14.25, p=.001 | F(1,18)=10.32, p=.005 | F(1,20)=9.36,  p=.006 | F(1,20)=13.63, p=.001 |
| Left rostral middle frontal cortex, BAI – functional connectivity correlation at post | ρ=-.65,  p<.001 | ρ=-.58,  p=.007 | ρ=-.57,  p=.005 | ρ=-.80,  p<.001 |
| Left rostral middle frontal cortex, BAI change – change in functional connectivity correlation | ρ=-.65,  p<.001 | ρ=-.71,  p=.001 | ρ=-.57,  p=.012 | ρ=-.73,  p=.001 |
| Right rostral middle frontal cortex, pre-post change in MBSR group | t(14)=-4.63,  p<.001 | t(9)=-3.91,  p=.004 | t(11)=-3.58,  p=.004 | t(11)=-4.76,  p=.001 |
| Right rostral middle frontal cortex, group by time interaction | F(1,24)=19.02,  p<.001 | F(1,18)=18.85,  p<.001 | F(1,20)=13.11, p=.002 | F(1,20)=19.90, p<.001 |
| Right rostral middle frontal cortex, BAI – functional connectivity correlation at post | ρ=-.57,  p=.002 | ρ=-.46,  p=.043 | ρ=-.58,  p=.005 | ρ=-.68,  p=.001 |
| Right rostral middle frontal cortex, BAI change – change in functional connectivity correlation | ρ=-.49,  p=.018 | ρ=-.51,  p=.026 | **ρ=-.32,  p=.179** | ρ=-.68,  p=.001 |
| Right superior frontal cortex, pre-post change in MBSR group | t(14)=-4.85,  p<.001 | t(9)=-4.56,  p=.001 | t(11)=-4.30,  p=.001 | t(11)=-4.70,  p=.001 |
| Right superior frontal cortex, group by time interaction | F(1,24)=25.72, p<.001 | F(1,18)=29.72, p<.001 | F(1,20)=20.99, p<.001 | F(1,20)=24.96, p<.001 |
| Right superior frontal cortex, BAI – functional connectivity correlation at post | ρ=-.47,  p=.015 | **ρ=-.30,  p=.204** | **ρ=-.41,  p=.059** | ρ=-.43,  p=.043 |
| Right superior frontal cortex, BAI change – change in functional connectivity correlation | ρ=-.42,  p=.044 | **ρ=-.42,  p=.074** | **ρ=-.33,  p=.173** | **ρ=-.44,  p=.058** |
